# Lipid rafts: a signaling platform linking cholesterol metabolism to synaptic deficits in autism spectrum disorders

**DOI:** 10.3389/fnbeh.2014.00104

**Published:** 2014-03-27

**Authors:** Hansen Wang

**Affiliations:** Faculty of Medicine, University of TorontoToronto, Ontario, Canada

**Keywords:** cholesterol, lipid rafts, autism spectrum disorders, synaptic plasticity, fragile X syndrome, Rett syndrome, FMRP, statins

## Autism and cholesterol metabolism

Autism spectrum disorders (ASDs) are a group of developmental psychiatric disorders characterized by impaired social interaction and communication, and by restricted, repetitive and stereotyped behaviors and interests (Zoghbi and Bear, 2012; Ebert and Greenberg, 2013; Huguet et al., 2013). Since the features of ASDs usually manifest at the early childhood when sensory experience is modifying the development and balance of excitatory and inhibitory synapses, it has been hypothesized that ASDs may be due to the disruption of experience dependent synaptic development and function, resulting in an imbalance between excitation and inhibition in brain (Auerbach et al., 2011; Zoghbi and Bear, 2012; Delorme et al., 2013; Wang and Doering, 2013). Although genetic causes have been identified in many individuals with ASDs, the details about how those causal genes converge on common pathways to alter synaptic homeostasis in ASDs still need to be investigated. Recently, Buchovecky et al. described disturbances in cholesterol homeostasis in animal model of the ASD Rett syndrome (RTT), giving rise to an exciting prospect that changes in cholesterol metabolism might underlie the development of ASDs (Buchovecky et al., 2013).

**Figure 1:**
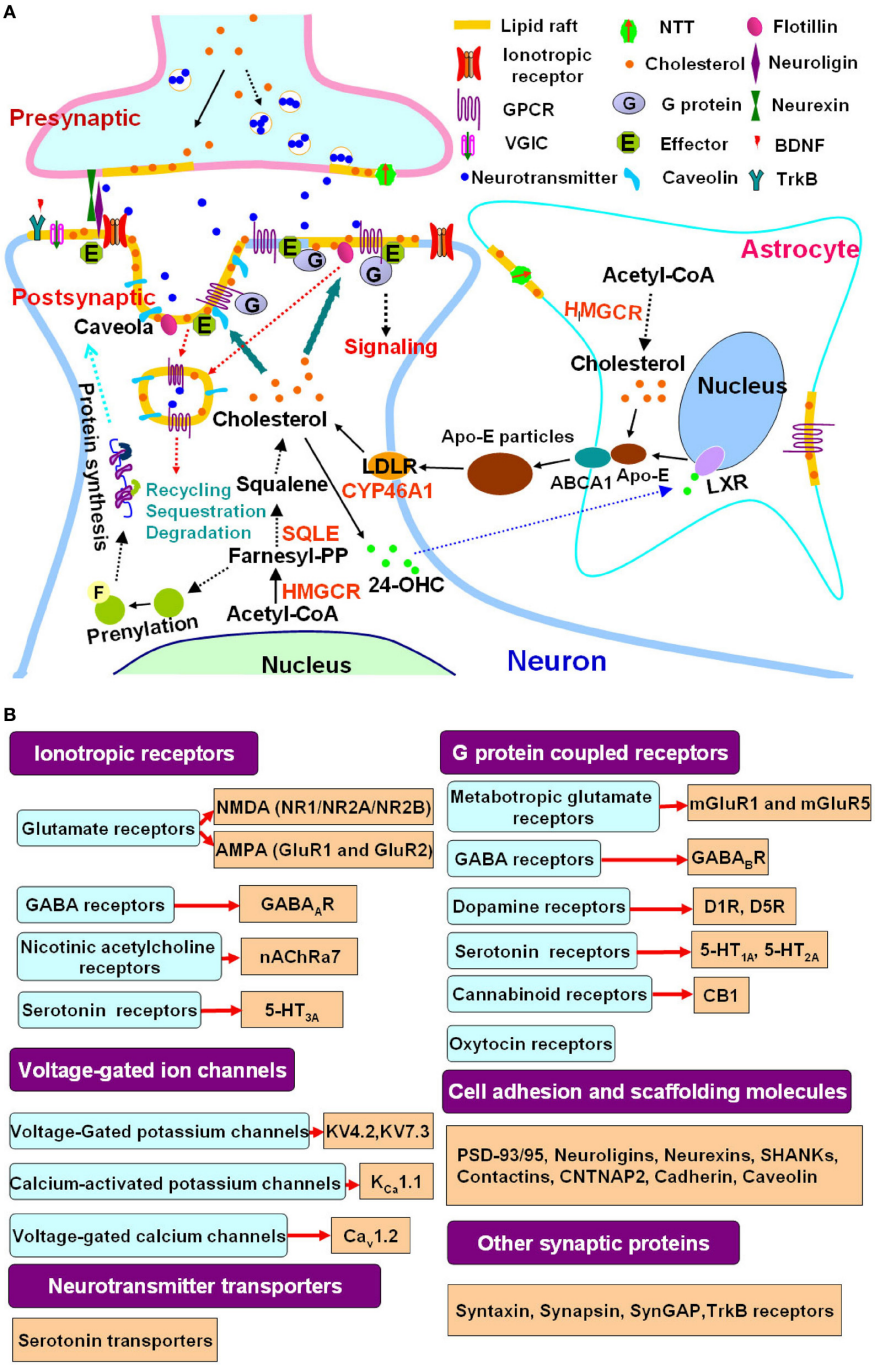
**Cholesterol metabolism, lipid rafts and autism spectrum disorders. (A)** Lipid rafts link cholesterol metabolism to synaptic signaling. Cellular cholesterol is synthesized from acetyl-CoA in a multistep pathway, with HMGCR and SQLE as the rate-limiting enzymes. In the adult brain, cholesterol synthesis is attenuated in neurons that rely on astrocyte derived cholesterol. Cholesterol and ApoE synthesized in astrocytes are secreted in an ABCA1 dependent process, forming discoidal lipoprotein particles. ApoE is a ligand for LDLR family members, which mediate neuronal lipoprotein uptake, thereby providing a supply of cholesterol to neurons. Excess cholesterol is hydroxylated by CYP46A1 to 24-OHC, which diffuses into the circulation. In astrocytes, 24-OHC binds to LXRs, which upregulate ApoE and ABCA1 expression. Cholesterol synthesis is also linked to the mevalonate pathway and produces Farnesyl-PP, which induces protein prenylation, a posttranslational modification that is important for the function of signaling proteins such as Ras and thus modulates protein synthesis. A mutated SQLE attenuates phenotypes of animal model of the ASD Rett syndrome (Buchovecky et al., 2013). Statins act at HMGCR and benefit animal models of Rett syndrome and other ASDs including fragile X syndrome and neurofibromatosis type 1 through inhibition of cholesterol synthesis and mevalonate pathway (Li et al., 2005; Buchovecky et al., 2013; Osterweil et al., 2013). Cholesterol is not evenly distributed in cell membranes. Lipid rafts are membrane microdomains enriched in cholesterol. A great number of presynaptic and postsynaptic proteins involved in neuronal communication are localized to lipid rafts. Lipid rafts are critical for neurotransmitter release, clustering of postsynaptic signaling molecules, protein trafficking and communication between the cell membrane and cytoplasma. They modulate the function of ionotropic receptors, G protein coupled receptors, voltage-gated ion channels as well as neurotransmitter transporters. Caveolae, a subset of lipid rafts that are flask-shaped membrane invaginations and contain caveolins, is involved in lipid raft protein recycling, sequestration and degradation (Allen et al., 2007; Pfrieger and Ungerer, 2011; Sebastiao et al., 2012). **(B)** Representative autism linked signaling proteins which are associated with lipid rafts. There is an increasing overlapping between affected signaling molecules or pathways in ASDs and lipid raft associated synaptic proteins as revealed by studies in autism genetics (Huguet et al., 2013; Murdoch and State, 2013; Persico and Napolioni, 2013; Schmunk and Gargus, 2013; Krumm et al., 2014; Ronemus et al., 2014) and neurobiology (Wang et al., 2008b, 2010; Zoghbi and Bear, 2012; Delorme et al., 2013; Ebert and Greenberg, 2013; Won et al., 2013) as well as research in lipid rafts (Allen et al., 2007; Korade and Kenworthy, 2008; Pristera and Okuse, 2011; Suzuki et al., 2011; Sebastiao et al., 2012; Liu et al., 2013). These studies further support the functional link between lipid rafts and synaptic deficits in ASDs. Abbreviations: 24-OHC, 24-hydroxycholesterol; ABCA1, ATP-binding cassette transporter A1; ApoE, apolipoprotein E; HMGCR, 3-hydroxy-3-methylglutaryl-CoA reductase; SQLE, squalene epoxidase; CYP46A1, cholesterol 24-hydroxylase; LDLR, LDL receptor; LXR, liver X receptor; Farnesyl-PP, farnesyl pyrophosphate; GPCR, G protein coupled receptors; VGIC, voltage-gated ion channels; NTT, neurotransmitter transporters, BDNF, brain-derived neurotrophic factor; PSD, postsynaptic density; ASD, autism spectrum disorder.

Cholesterol, an essential cell membrane component, influences the establishment and maintenance of synaptic connection and glial cell development in the nervous system. Balanced cholesterol homeostasis is an important aspect of nervous system function (Mauch et al., 2001; Pfrieger, 2003; Linetti et al., 2010; Pfrieger and Ungerer, 2011; Mathews et al., 2014) (Figure 1A). Perturbed cholesterol homeostasis can affect neural development and synaptogenesis and result in synaptic dysfunction, thus may lead to disorders of nervous system (Simons and Ehehalt, 2002; Linetti et al., 2010; Pani et al., 2010; Karasinska and Hayden, 2011). Evidence from patients indicates that cholesterol homeostasis could be altered in autistic disorders. The Smith-Lemli-Opitz Syndrome (SLOS), a genetic condition of impaired cholesterol biosynthesis due to mutations of the 7-dehydrocholesterol reductase gene (*DHCR7*), has been found to be associated with autism, supporting genetic defects in cholesterol metabolism can cause autism (Sikora et al., 2006; Bukelis et al., 2007; Diaz-Stransky and Tierney, 2012). Abnormal cholesterol metabolism has also been observed in patients with the ASD Asperger syndrome and other nonsyndromic ASDs, suggesting different abnormalities of cholesterol metabolism may exist in ASDs (Tierney et al., 2006; Dziobek et al., 2007). However, little is known about the mechanisms that mediate the abnormal cholesterol metabolism in these ASD conditions.

RTT is caused largely by mutations in the X-linked methyl CpG-binding protein 2 gene (*MECP2*) (Baker et al., 2013; Lyst et al., 2013; Xu and Pozzo-Miller, 2013). In *Mecp2* mutant mice, Buchovecky et al. found that the expression of genes (*Hmgcr, Sqle* and *Cyp46a1*) which encode key enzymes (3-hydroxy-3-methylglutaryl-CoA reductase, squalene epoxidase, CYP46A1) in the cholesterol metabolic pathway and cholesterol concentrations are altered in the brain in a development dependent manner (Buchovecky et al., 2013). Their study indicates that loss of Mecp2 disrupts cholesterol homeostasis, suggesting abnormal cholesterol metabolism might be involved in the pathogenesis of RTT (Nagy and Ackerman, 2013). It thus links the autism associated gene to cholesterol metabolism, providing further insights into the relationship between cholesterol metabolism and ASDs.

## Cholesterol, lipid rafts and synaptic plasticity

Disturbances in cholesterol homeostasis in ASDs raise the question of how changes in cholesterol metabolism might be involved in the development of autism. Cholesterol is not uniformly distributed in biological membranes, but concentrated in lipid rafts, together with other lipids like sphingolipids (Pfrieger, 2003; Fielding and Fielding, 2004; Korade and Kenworthy, 2008; Pfrieger and Ungerer, 2011). Lipid rafts, which are believed to act as platforms for cellular signal transduction, have been shown to play roles in synaptic plasticity and contribute to neuropathologies, such as Alzheimer's disease (AD), Parkinson's disease (PD), Huntington disease (HD) (Tsui-Pierchala et al., 2002; Pfrieger, 2003; Korade and Kenworthy, 2008; Pani et al., 2010; Karasinska and Hayden, 2011; Sebastiao et al., 2012). Cholesterol is a key component of lipid rafts; its depletion disrupts lipid rafts and leads to synaptic dysfunction or loss of synapses (Mauch et al., 2001; Fielding and Fielding, 2004; Korade and Kenworthy, 2008; Linetti et al., 2010; Pfrieger and Ungerer, 2011; Pristera and Okuse, 2011; Sebastiao et al., 2012). Since altered synaptic function is the common basis for ASDs (Santoro et al., 2012; Zoghbi and Bear, 2012; Delorme et al., 2013; Ebert and Greenberg, 2013; Wang and Doering, 2013), it is likely that abnormal cholesterol metabolism might be implicated in autism through lipid raft disarrangements.

Lipid rafts are specialized membrane structures that form an organized portion of the membrane with concentrated signaling molecules and link to the cytoskeleton (Simons and Toomre, 2000; Simons and Ehehalt, 2002; Tsui-Pierchala et al., 2002; Fielding and Fielding, 2004; Allen et al., 2007; Korade and Kenworthy, 2008; Lingwood and Simons, 2010; Simons and Sampaio, 2011). The synapses, both the presynaptic and postsynaptic sites, are highly enriched in lipid rafts. Lipid rafts not only contribute to neurotransmitter exocytosis in presynaptic terminals, but also postsynapticly modulate neuronal signaling through clustering of neurotransmitter receptors, ion channels and components of downstream effectors (Tsui-Pierchala et al., 2002; Korade and Kenworthy, 2008; Linetti et al., 2010; Pfrieger and Ungerer, 2011; Pristera and Okuse, 2011; Sebastiao et al., 2012) (Figure 1A). The postsynaptic density (PSD), which is a massive multi-protein complex whose functions include positioning signaling molecules for synaptic plasticity, could be physically associated with lipid rafts (Hering et al., 2003; Sheng and Hoogenraad, 2007; Delint-Ramirez et al., 2010; Sheng and Kim, 2011; Suzuki et al., 2011; Liu et al., 2013). Numerous PSD proteins such as NMDA receptors (NR1, NR2A and NR2B), AMPA receptors (GluR1 and GluR2), metabotropic glutamate receptors (mGluRs), PSD-93/95, CaMKII, and cadherin, are associated with synaptic lipid rafts; notably, PSD-95 binds to the postsynaptic neuroligins which interact with the presynaptic neurexins, providing a transsynaptic link between PSD and presynaptic active zone (Hering et al., 2003; Delint-Ramirez et al., 2010; Sheng and Kim, 2011; Suzuki et al., 2011; Liu et al., 2013). The association of PSD with lipid rafts might be important in signal integration and synaptic function. Accumulating evidence has indicated that precise localization of neurotransmitter receptors, transporters, ion channels and other synaptic proteins in lipid rafts can be regulated by cholesterol and this regulation is critical for synaptic plasticity (Tsui-Pierchala et al., 2002; Hering et al., 2003; Allen et al., 2007; Linetti et al., 2010; Pfrieger and Ungerer, 2011; Pristera and Okuse, 2011; Sebastiao et al., 2012) (Figure 1A). Importantly, many of the lipid rafts associated signaling molecules or pathways have been found to be implicated in autism (Tsui-Pierchala et al., 2002; Allen et al., 2007; Pristera and Okuse, 2011; Sebastiao et al., 2012; Zoghbi and Bear, 2012; Delorme et al., 2013; Ebert and Greenberg, 2013; Huguet et al., 2013; Schmunk and Gargus, 2013; Won et al., 2013) (Summarized in Figure 1B). Therefore, it is reasonable to postulate that lipid rafts might serve as a platform where cholesterol imbalance eventually causes neuronal and synaptic deficits in ASDs (Wassif et al., 2001; Waage-Baudet et al., 2005; Lee and Tierney, 2011).

## Cholesterol, lipid rafts and ASD therapy

Interestingly, genetic or pharmacological manipulation of cholesterol metabolism has been found to alleviate ASD associated animal behaviors. In the aforementioned study, a mutagenesis screen in *Mecp2* mutant mice has identified that a nonsense mutation in SQLE, which is an obligate rate-limiting enzyme in cholesterol biosynthesis, diminished some of RTT related phenotypes (Buchovecky et al., 2013). Statins reduce cholesterol synthesis by interfering with cholesterol synthesis through competitive inhibition of HMGCR. Since cholesterol synthesis is functionally tied to the mevalonate pathway, statins also inhibit the synthesis of isoprenoid intermediates farnesyl pyrophosphate and ubiquinones, and thus affect protein modifications such as prenylation, which are important for the localization and function of signaling proteins such as Ras (van der Most et al., 2009; Nagy and Ackerman, 2013). Due to their impact on cholesterol synthesis and protein modification, statins perturb the composition and properties of lipid rafts (van der Most et al., 2009). These drugs might exert effect on RTT animals as manipulation of SQLE did. As expected, treatment with statins did ameliorate metabolic symptoms and improved animal motor coordination and activity of the *Mecp2* mutant mice (Buchovecky et al., 2013).

Noteworthily, the effect of statin treatment has also been observed in animal models of other ASDs, including fragile X syndrome and neurofibromatosis type 1 (Li et al., 2005; Osterweil et al., 2013). Group I mGluRs (mGluR1 and mGluR5) are present in lipid rafts and play critical roles in activity dependent synaptic plasticity (Sebastiao et al., 2012; Wang and Zhuo, 2012). In animal models of fragile X syndrome, the activity of group I mGluRs is abnormally enhanced, which is causally linked to the pathogenesis of this disease condition (Bear et al., 2004; Wang et al., 2008a; Santoro et al., 2012; Zoghbi and Bear, 2012). The scaffolding protein caveolin in lipid rafts, alteration in membrane cholesterol content or perturbation of lipid rafts have been shown to be able to regulate group I mGluR trafficking and downstream signaling pathways such as mitogen-activated protein Kinases (MAPKs) (Francesconi et al., 2009; Kumari et al., 2013). In cultured neurons from fragile X mice, group I mGluR dependent signaling was attenuated by statins (Kumari et al., 2013). Consistently, application of statins reduced abnormally elevated protein synthesis and mGluR-long term depression (LTD) in hippocampal slices of fragile X mice, attenuated audiogenic seizures and corrected the hyperexcitability in the visual cortex of these animals (Osterweil et al., 2013). Apart from inhibition of signaling molecules such as Ras signaling for protein synthesis which has been suggested by the investigators to underlie the observed effects (Li et al., 2005; Osterweil et al., 2013), statins may also benefit ASD conditions through their action on the cholesterol-lipid raft-mGluR pathway. Taken together, these studies further support defective cholesterol homeostasis may underlie the ASD phenotypes, suggesting that cholesterol metabolism and lipid rafts could be therapeutic targets for ASDs. Considering the complexity and heterogeneity of autism, it will be necessary to assess in future studies the effect of statin treatment on the full spectrum of ASD phenotypes.

## Challenges and open questions

It is important to note, cholesterol may regulate protein function not only through altering lipid rafts but also by direct interactions with the proteins. However, it is very challenging to discriminate between these possibilities for any specific type of protein because it is unreasonable to test the effect of cholesterol on the function of a membrane protein in an environment without lipid rafts (Levitan et al., 2010). Since cholesterol metabolism in brain depends on the cooperation between neurons and astrocytes, it will be necessary to examine or manipulate cholesterol metabolism and lipid rafts in individual cell types (neurons or glial cells) of the diseased brain (Korade and Kenworthy, 2008; Pfrieger and Ungerer, 2011). In view of that lipid rafts could be potentially modifiable by dietary cholesterol, gangliosides, and fatty acids (Yaqoob and Shaikh, 2010), the relevance and significance of those dietary supplements for ASD treatment remains to be determined. Although statins have shown to be promising for ASD therapy, the timing, dosage and ideal drugs will need to be identified in animal models or other preclinical trials to facilitate the design of individualized therapeutic strategy (Buchovecky et al., 2013; Nagy and Ackerman, 2013; Osterweil et al., 2013).

Despite the controversies in lipid raft research, it is now clear that lipid rafts which represent dynamic structural components of cellular membranes do play roles in neuronal signaling and that dysregulation of lipid rafts can lead to diseases (Allen et al., 2007; Korade and Kenworthy, 2008; Pfrieger and Ungerer, 2011; Pristera and Okuse, 2011; Sebastiao et al., 2012). Due to complexity of ASD pathology and etiology, the involvement of lipid rafts in these disease conditions is not simple. The disturbed cholesterol metabolism or lipid raft aberrance may not exist in all cases or every stage of the ASDs. Thus, lipid rafts should not be seen as the simplistic link between genetic defects and synaptic dysfunction in autism. Additional studies are definitely required to investigate cholesterol metabolism and lipid raft abnormalities in ASDs and their relevance to the pathophysiology and treatment of these neurodevelopmental disorders. These studies will need to combine biochemical, biophysical, imaging, behavioral, genetic and pharmacological techniques to advance our understanding and therapeutics of autism.
